# Mitochondria-ER contact site components regulate the formation and localization of specialized high-capacity mitochondria in the *C. elegans* anchor cell.

**DOI:** 10.17912/micropub.biology.001679

**Published:** 2025-08-11

**Authors:** Isabel W. Kenny-Ganzert, David R. Sherwood

**Affiliations:** 1 Department of Biology, Duke University, Durham, NC USA

## Abstract

Mitochondria-endoplasmic reticulum contact sites (MERCS) play crucial roles in mediating calcium signaling and lipid metabolism, and regulate mitochondrial morphology, function, and quality control. Recent studies have found that the *C. elegans* anchor cell (AC) harbors a specialized pool of high-capacity mitochondria that localize to the invasive front and are enriched with electron transport chain proteins to generate high ATP levels to fuel invasion. We conducted an RNAi screen of 59 MERCS-encoding components and identified over 30 required for high-capacity mitochondria formation. Our results suggest that MERCS may play a key role in the formation of specialized high-capacity mitochondria.

**Figure 1. Mitochondria-ER contact site components regulate high-capacity mitochondria formation and localization within the C. elegans anchor cell.**
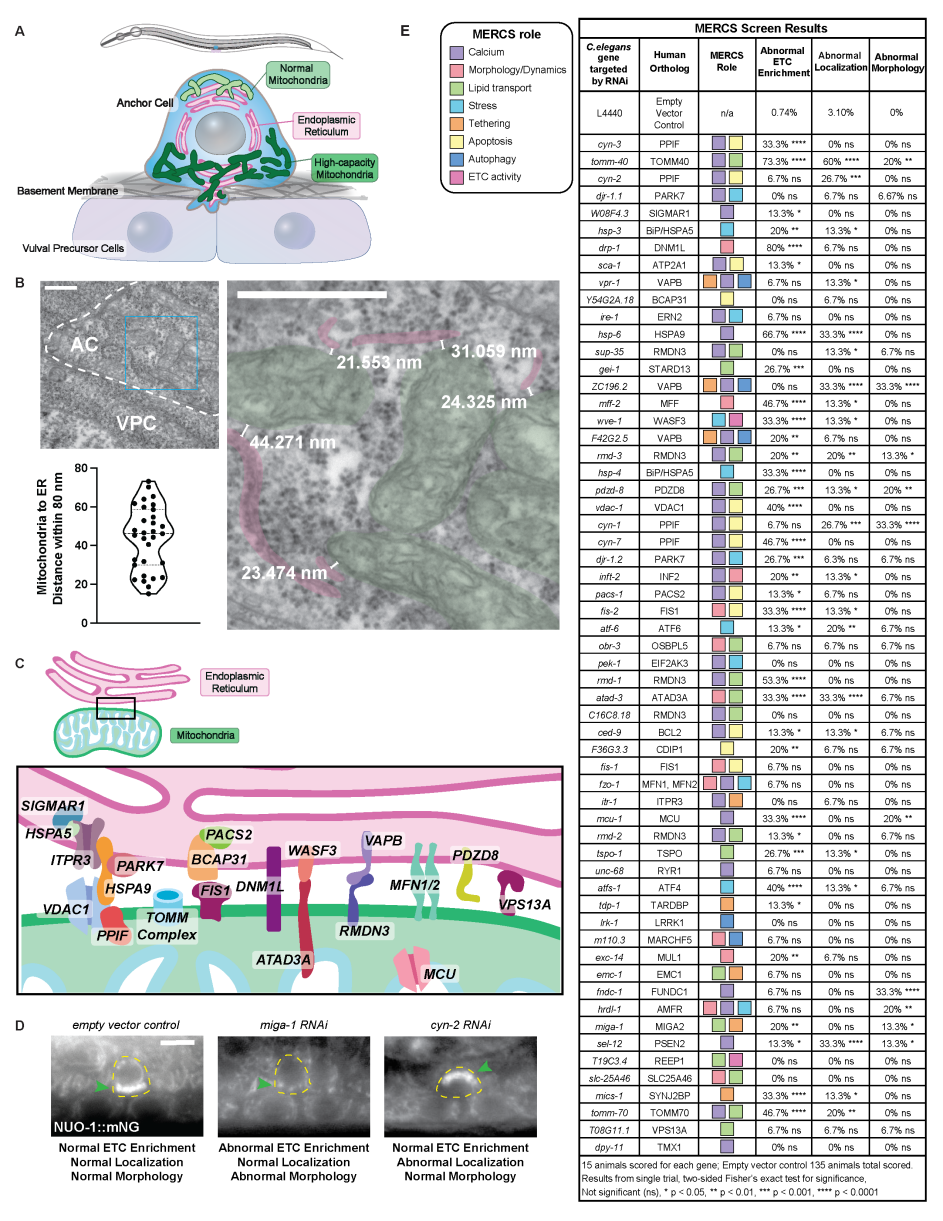
**A)** At the L3 larval stage (top) the anchor cell (AC, blue) invades through the basement membrane (gray) to contact the vulval precursor cells (VPCs, light purple). Previous work has shown that the AC contains an extensive endoplasmic reticular (ER) network (pink) that enters the invasive protrusion (Park et al., 2024) and ETC-enriched high-capacity mitochondria that localize to the invasive front (dark green) and are a distinct population from apical lower ETC-enriched mitochondria (light green) that are similar in composition to neighboring non-invasive cells. **(B)** (Left top) Transmission electron microscopy image of a thin section cut through the AC (dotted outline) above the VPC prior to invasion. (Right) Enlarged area of the AC corresponding to the blue box in the left panel shows where mitochondria (green) are near ER (pink). Distance (nm) measured illustrated by white bracketed line. Scale bars, 500 nm. (Bottom left) Violin plot of mitochondria to ER distance measured in mitochondria within 80 nm of ER (n = 31 mitochondria in 5 ACs). **(C)** Schematic illustration of many of the proteins with known or predicted roles in mitochondria-ER contact sites (MERCS). SIGMAR1, sigma non-opioid intracellular receptor 1 (calcium regulation); HSPA5, also known as BiP, heat shock protein family A (Hsp70) member 5 (stress); ITPR3, inositol 1,4,5-trisphosphate receptor type 3 (calcium regulation, tethering); PARK7, Parkinsonism associated deglycase (calcium regulation, stress); HSPA9, heat shock protein family A (Hsp70) member 9 (calcium regulation); VDAC1, voltage dependent anion channel 1 (calcium regulation, apoptosis); PPIF, peptidylprolyl isomerase F (calcium regulation, apoptosis); TOM Complex, translocase of outer mitochondrial membrane (calcium regulation, lipid transport); PACS2, phosphofurin acidic cluster sorting protein 2 (calcium regulation, apoptosis); BCAP31, B cell receptor associated protein 31 (apoptosis); FIS1, fission, mitochondrial 1 (morphology/dynamics, apoptosis); DNM1L, also known as DRP1, dynamin 1 like (morphology/dynamics); ATAD3A, ATPase family AAA domain containing 3A (lipid transport, ETC activity); WASF3, WASP family member 3, (stress, ETC activity); RMDN3, regulator of microtubule dynamics 3 (calcium regulation, lipid transport); VAPB, VAMP associated protein B and C (calcium regulation, tethering, autophagy); MFN1/2, mitofusin 1 and 2 (morphology/dynamics, calcium regulation, stress); PDZD8, PDZ domain containing 8 (calcium regulation, lipid transport); VPS13A, vacuolar protein sorting 13 homolog A (lipid transport). **(D)** Examples of phenotypes observed after RNAi targeting MERCS genes. (Left) Normal ETC enrichment (endogenously tagged NUO-1::mNG, arrowhead) in AC (yellow dashed line) high-capacity mitochondria localized to the invasive front treated with empty vector control RNAi. (Middle) Reduced ETC (NUO-1::mNG) enrichment, normal invasive localization, and abnormal morphology where mitochondria are more punctate and fail to form a branched network (arrowhead) after RNAi targeting *miga-1* . (Right) Normal ETC enrichment (NUO-1::mNG), normal morphology, but abnormal localization where most of the ETC-enriched mitochondria apically localized (arrowhead) after RNAi targeting *cyn-2* . Scale bar, 5 μm. **(E)** MERCS screen results.

## Description

Cell invasion through basement membrane (BM) extracellular matrices (ECMs) is important in development, immune surveillance, and is dysregulated in many diseases, such as cancer (Kenny-Ganzert & Sherwood, 2024). BMs are a highly dense type of ECM. The membrane protrusions used by invasive cells to break through BMs are energetically intensive and require high ATP levels to fuel F-actin turnover, dynamic protease delivery, and active membrane trafficking (Li et al., 2019; Marshansky & Futai, 2008; Shah et al., 2015). Emerging evidence indicates that mitochondria and oxidative phosphorylation (OXPHOS) play a crucial role in delivering ATP to fuel invasive protrusions (Garde & Sherwood, 2021). An excellent model to study cell invasion through BM is the *C. elegans* anchor cell (AC). The AC is a specialized uterine cell that invades through BM during an ~90-minute window during the L3 larval stage to connect the uterine and vulval tissues to form the egg-laying apparatus (Kenny-Ganzert & Sherwood, 2024). Recent studies in our group have revealed that the *C. elegans* anchor cell (AC) relies upon specialized high-capacity mitochondria that are enriched with electron transport chain (ETC) proteins found in the folds (cristae) of the inner mitochondrial membrane (Kenny-Ganzert et al., 2025). The ETC transfers electrons through a series of protein complexes to form a proton gradient that drives ATP synthesis. These high-capacity mitochondria are concentrated at the AC's invasive front, where they provide a localized source of ATP to fuel invasion ( Figure 1A ) (Garde et al., 2022; Kenny-Ganzert et al., 2025). We have found that high-capacity mitochondria are specified early by the AC's pro-invasive transcriptional program, and that they require dense cristae formation and increased mitochondrial protein import machinery. We further discovered that netrin signaling through a Src family kinase directs microtubule polarization, which facilitates metaxin adaptor complex dependent high-capacity mitochondrial trafficking to the site of invasive protrusion formation (Kenny-Ganzert et al., 2025).

Mitochondria transiently connect and interact with the endoplasmic reticulum (ER) (Aoyama-Ishiwatari & Hirabayashi, 2021). The contact sites between the outer mitochondrial membrane and the ER are referred to as mitochondria–ER contact sites (MERCS). These interorganellar junctions are enriched in specific lipids and specialized proteins that define their structure and functions (Larrañaga-SanMiguel et al., 2025; Sassano et al., 2022). MERCS serve as key sites for lipid transfer and calcium (Ca ^2+^ ) signaling, and have functions in cellular bioenergetics, proteostasis, mitochondrial quality control, mitochondrial morphology/dynamics, apoptosis and autophagy (Flis & Daum, 2013; Giacomello & Pellegrini, 2016; Gómez-Suaga et al., 2019; Lewis et al., 2016; Loncke et al., 2021; Nakamura et al., 2025; Namba, 2019). With our recent discovery of specialized high-capacity mitochondria, we wanted to determine if MERCS might have a role in their formation and invasive enrichment.

MERCS form between juxtaposed mitochondria and ER membranes that are ~10 – 80 nm apart (Giacomello & Pellegrini, 2016; Larrañaga-SanMiguel et al., 2025). Transmission electron microscopy (TEM) can be used to identify MERCS, although the thin 2D sectioning is limited in detecting most MERCS due to their dynamic nature, as MERCS rapidly form and disassemble (Bernhard & Rouiller, 1956; Giamogante et al., 2020; Larrañaga-SanMiguel et al., 2025). Using thin sectioned (35 – 50 nm) transmission electron microscopy images taken from five ACs (Hall et al., 2012; Morrissey et al., 2014), we examined 50 mitochondria within the basal, invasive side of the AC, and found 31 were within 80 nm of ER. Of these seven mitochondria were in possible contact with the ER (see Methods). These results are consistent with the possibility of MERCS formation in the AC. The average distance between AC mitochondria and ER in the 31 cases was ~44 nm ( Figure 1B ). However, the distance between mitochondria and ER at the site of known MERCS formation was determined in mammalian cells (Giamogante et al., 2020; Larrañaga-SanMiguel et al., 2025). As the dimensions and arrangement of mitochondria within mammalian cells can differ from *C. elegans '* mitochondria (Mondal et al., 2021; Riboul et al., 2024), it is possible that the distances are distinct in *C. elegans* . Mitochondria-ER contact sites are maintained and regulated by many protein interactions, some of which are thought to function as physical tethers, whereas others have roles in mediating calcium and lipid transport between the juxtaposed organelles ( Figure 1C ) (Aoyama-Ishiwatari & Hirabayashi, 2021; Ellenrieder et al., 2019). Notably, the proteins that make up these contact sites are neither exclusive nor specific to MERCS. This presents challenges in delineating specific roles for the MERCS versus functions independent of MERCS (Larrañaga-SanMiguel et al., 2025). To test the possible roles of MERCS in AC mitochondria, we compiled a list of 59 genes with either known or predicted roles in MERCS and assessed if RNAi-mediated knockdown of these genes affected high-capacity mitochondria ETC enrichment, invasive location, and morphology, as mitochondrial morphology can affect ATP output and transport (Aoyama-Ishiwatari & Hirabayashi, 2021; Carreras-Sureda et al., 2019; Chami et al., 2008; Chen et al., 2023; Diokmetzidou & Scorrano, 2025; Galmes et al., 2016; Hinton et al., 2024; Janer et al., 2016; Larsen et al., 2020; Liu et al., 2019; Moras et al., 2020; Morris et al., 2021; Paillusson et al., 2016; Puebla-Huerta et al., 2025; Toyofuku et al., 2020; Tubbs & Rieusset, 2017; Weng et al., 2017; Ziegler et al., 2021) ( Figure 1D- E).

We categorized the MERCS genes screened based on the following criteria: **Calcium** – regulates or facilitates calcium signaling between the ER and mitochondria; **Morphology/Dynamics** – regulates or is directly required for mitochondrial fission or fusion; **Lipid Transport** – regulates or facilitates phospholipid or cholesterol transport between the ER and mitochondria; **Stress** – induced by or responds to mitochondrial and ER stress; **Tethering** – physically links the mitochondria and ER; **Apoptosis** – regulates or is required for ER-signaled apoptosis; **Autophagy** – regulates or is required for autophagy; and **ETC activity** – mitochondria-ER contact that is known to affect ETC activity. We found that RNAi-mediated reduction of 36 of the 59 genes reduced mitochondrial ETC enrichment, 23 perturbed mitochondrial localization, and 8 affected mitochondrial morphology. The genes that had the greatest effect on ETC enrichment, localization, and morphology were involved in calcium and/or lipid transport: of the 43 genes that affected at least one of the high-capacity mitochondria phenotypes, 25 were involved in calcium regulation and 11 in lipid transport. Interestingly, multiple genes that regulate mitochondrial fission and fusion – *drp-1* , *fis-1* , and *fzo-1* , did not appear to affect mitochondrial morphology. It is possible, however, that we did not detect more subtle morphology changes due to the limited resolution of compound microscopy. Knockdown of four genes – *tomm-40* , *pdzd-8* , *rmd-3* , and *sel-12* – affected all phenotypes examined and all four genes are involved in calcium regulation, lipid transport or both. TOMM-40 (human TOMM40) is part of the translocase complex in the outer mitochondrial membrane and has a direct role in calcium signaling from the ER, as well as an indirect role in cholesterol transport from the ER (Yang et al., 2024). PDZD-8 (human PDZD8) is an ER-resident protein that is required for calcium to be released from the ER into the mitochondria (Hirabayashi et al., 2017). RMD-3 (human RMDN3) is a mitochondrial protein that regulates calcium homeostasis, phospholipid transfer, and mitochondrial oxidative stress (De Vos et al., 2012; Ito et al., 2022; Shiiba et al., 2025). SEL-12 (human PSEN2) is an ER-resident protein that regulates calcium transport from the ER to mitochondria (Sammeta et al., 2023). The penetrant and multifaceted mitochondrial defects observed after RNAi-mediated reduction of these MERCS proteins suggests that these genes have broad regulatory roles in promoting the formation and localization of the AC's high-capacity mitochondria.

In summary, we found that AC mitochondria are in close enough proximity to form MERCS and RNAi-mediated knockdown of many MERCS encoding genes affect high-capacity mitochondrial ETC enrichment, morphology, and localization. This work lays the foundation for future studies directly examining the formation, composition and function of MERCS in the AC and deeper analysis of genes encoding MERCS components and their roles in promoting high-capacity mitochondria formation and function.

## Methods

*Caenorhabditis elegans* were maintained at 20°C on nematode growth media (NGM) plates seeded with *Escherichia coli* strain OP50 . The electron microscopy images used to measure the distance between juxtaposed mitochondrial and endoplasmic reticular membranes are from a depositary of images from previously published work (Morrissey et al., 2014). 50 mitochondria from five ACs (5-14 mitochondria from each AC) were selected based on clear edges of mitochondrial membrane. If the mitochondrial and ER membrane were clearly apparent and juxtaposed, the “straight freehand line” tool in Fiji/ImageJ was then used to measure the distance between the mitochondrion and closest ER. If the distance between mitochondrion and ER was less than 80 nm, the distance was recorded as a possible site where mitochondria-ER contact could form. Further, in cases where the mitochondria and ER were within 25 nm, it was scored as a possible direct contact given the resolution of the TEM images. If the distance was greater than 80 nm or there was no apparent ER in the vicinity of that mitochondrion, it was not counted as a site where mitochondria-ER contact could form. The MERCS screen was performed using RNAi clones from either the Vidal or Ahringer feeding RNAi libraries, which were streaked onto LB plates containing tetracycline and ampicillin. For the MERCS screen, RNAi clones from the Ahringer feeding library were used for the following genes: *atad-3 , C16C8.18 , ced-9 , F36G3.3 , fis-1 , fzo-1 , itr-1 , mcu-1 , rmd-2 , tspo-1 , unc-68 , emc-1 , hrdl-1 , T08G11.1* . The Vidal feeding library was used for the others. For RNAi screening, a single colony was inoculated in liquid LB plus 100 μL/mL ampicillin, then placed in a 37°C shaker for growth overnight. The double-stranded RNA (dsRNA) was induced by adding 1 μL/mL IPTG followed by shaking for an additional hour before plating onto NGM plates with IPTG and ampicillin. Screening was performed using *C. elegans* strain NK2657, which have the ETC protein NUO-1 endogenously tagged with mNeonGreen (mNG) fluorescent protein and AC-specific expression of the moesin actin-binding domain (ABD) fused to mCherry. The AC-specific mCherry was used to confirm the identity of the AC. Animals were synchronized using a standard hypochlorite synchronization protocol (Porta-de-la-Riva et al., 2012), plated at L1 larval stage , and grown for 36-40 hours until the P6.p vulval precursor cell (VPC) beneath the AC had undergone one division (referred to as the P6.p 2-cell stage). P6.p 2-cell stage animals were mounted on 5% agar pads with 1% sodium azide to anesthetize the worms and imaged on a Zeiss upright compound microscope equipped with 488 and 561 nm filters and a Nomarski prism for differential interference contrast (DIC). A total of 15 animals were scored by eye for each gene targeted by RNAi to determine if the RNAi treatment resulted in altered ETC enrichment, morphology, and/or localization. In brief, animals were scored by eye using the following criteria: (1) if the AC was not immediately apparent compared to neighboring uterine cells based on the NUO-1::mNG enrichment, then it was scored as having abnormal ETC enrichment; (2) If greater than 50% of the mitochondria were not basally localized, it was scored as abnormal localization, (3) If the greater than 25% of the mitochondria appeared excessively punctate, it was scored as having abnormal morphology. For statistical analysis GraphPad Prism was used to conduct a two-sided Fisher's exact test to determine significance compared to the empty vector control. The schematics were drawn, and the figure was assembled in Adobe Illustrator.

## Reagents

**Table:** 

Reagent	Genotype/Description	Available From
*C. elegans* strain NK2657 (Kenny-Ganzert et al., 2025)	*nuo-1 (qy143[nuo-1::mNG]) II; qyIs50 [cdh-3p::moeABD::mCherry] *	CGC or by contacting david.sherwood@duke.edu
Vidal RNAi library (Rual et al., 2004)	Feeding RNAi library	Horizons Discovery
Ahringer RNAi library (Kamath et al., 2003)	Feeding RNAi library	DNAFORM
*E. coli* strain OP50	Standard food	CGC
